# Kikuchi-Fujimoto disease following SARS CoV2 vaccination: Case report

**DOI:** 10.1016/j.idcr.2021.e01253

**Published:** 2021-08-10

**Authors:** Hussam Al Soub, Wanis Ibrahim, Muna Al Maslamani, Gawahir A. Ali, Waseem Ummer, Ala’ Abu-Dayeh

**Affiliations:** a Department of Medicine, Hamad Medical Corporation, Doha, Qatar; b Department of Laboratory Medicine and Pathology, Hamad Medical Corporation, Doha, Qatar

**Keywords:** SARS CoV 2, Kikuchi, Fujimoto, Lymphadenopathy, COVID 19 vaccine

## Abstract

Kikuchi's disease (KD) also known as Kikuchi-Fujimoto disease (KFD), or histiocytic necrotizing lymphadenitis was first described in 1972 independently by Kikuchi and Fujimoto et al. It is a benign self-limited condition of unknown etiology which usually presents with cervical lymphadenopathy or fever of unknown origin. The diagnosis of KFD is based on histopathologic examination of the involved lymph node, showing the presence of well-defined necrosis without granulocytic cells. There is no special treatment for KFD. However non-steroidal anti-inflammatory drugs or corticosteroids are required occasionally to control the associated systemic manifestations. The outcome of the disease is usually favorable with resolution of symptoms in most cases within one to four months. We report a case of Kikuchi-Fujimoto disease that occurred in a young Qatari male patient 10 days following receiving the first dose of BNT162b2 vaccine. Diagnosis was established by lymph node biopsy and recovery was complete after 10 days.

## Case history

An 18-year-old Qatari male, presented in with fever of two weeks duration, not associated with chills, rigors or night sweats. He also complained of left sided neck swelling, decrease oral intake and nausea for the past 2 weeks. No complaints of cough, sore throat and no shortness breath. He denied history of contact with sick patient, recent travel, or contact with animals. He reports receiving the first dose SARS CoV2 vaccine (BNT162b2) 10 days before the start of his symptoms. His past history was remarkable for steroid dependent minimal change renal disease diagnosed in 2015. He was treated initially with prednisolone and cyclosporine for more than two years. He also received rituximab three times during 2018 and 2019 for relapse of his disease, however he was in remission for the last two years and not receiving any medications. He was non-smoker and non-alcohol drinker and works as a policeman. Physical examination on admission was unremarkable except for a temperature of 39 °C and the presence of multiple tender enlarged left cervical lymph nodes of varying size with the largest about 1.5 cm in diameter.

**Fig. 1:**
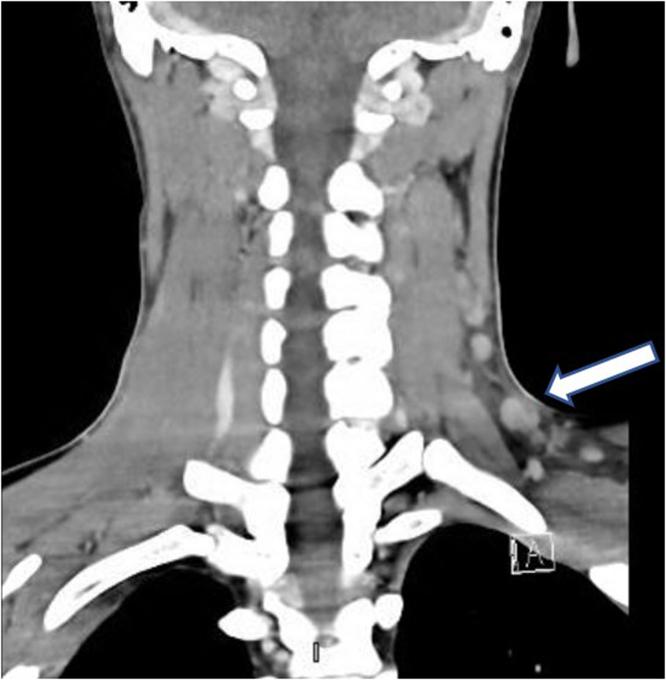
CT of the neck demonstrating left multiples supraclavicular lymphadenopathy the largest measured 10 × 11 mm (arrowed).

**Fig. 2:**
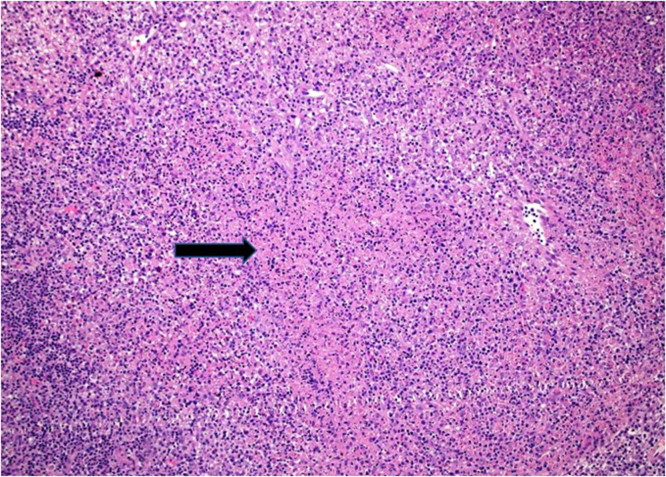
A low magnification H&E showing partial effacement of the lymph node architecture by pale necrotic foci.

**Fig. 3:**
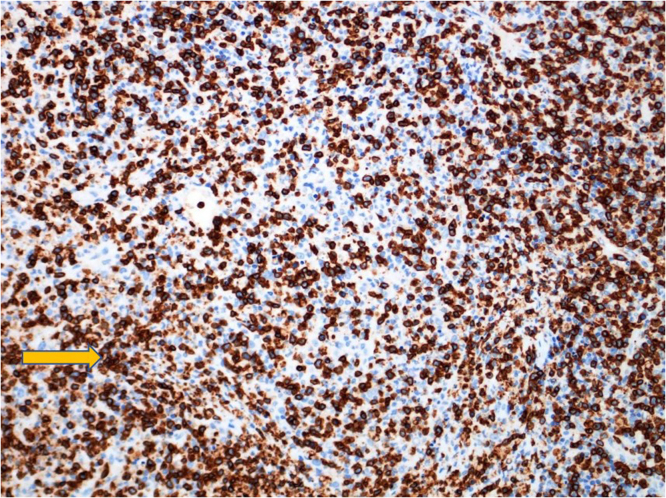
CD3 immunohistochemical stain highlights T-cells.

**Fig. 4:**
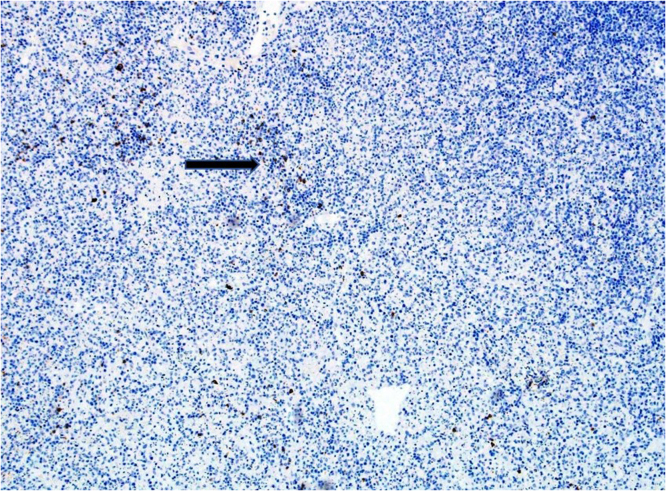
CD20 immunohistochemical stain highlights residual B-cells, while T-cells are negative for CD20.

Laboratory investigation on admission revealed, white blood cells (WBC) 2400/µL ( Neutrophils 1400 /µL, Lymphocytes 800 /µL, monocyte 200 /µL), hemoglobin (HB) 12.9 gm/dL, platelets 102,000 /µL, creatinine 70 µmol/L, CRP 38.4 mg/L, procalcitonin 0.46 ng/ml, ALT 64 U/L, AST 72 U/L, LDH 432 U/L, ferritin 699 µg/L, with negative ANA and normal C3, C4. Serology for HBV and HCV viruses were negative. HIV combo test and HIV PCR were negative. PCR for CMV and EBV were all negative. Blood and urine cultures were also negative. QuantiFERON TB Gold Plus was negative. Nasopharyngeal swab for SARS CoV 2 PCR was negative. Computed tomographic scan for chest and neck revealed multiple left cervical and axillary lymph nodes the largest one in the left supraclavicular region measuring around 10 × 11 mm (Fig. 1). Patient was initially treated with ceftriaxone however it was discontinued later on when the results of blood and urine were reported negative. He received symptomatic treatment with paracetamol and NSAIDS to control his fever. Excisional lymph node biopsy was done. Sections from the lymph node show effaced lymph node architecture by diffuse polymorphic lymphohistiocytic infiltrate with multiple foci of necrosis (Fig. 2). Many immunoblasts and numerous apoptotic bodies were present. Neutrophils were not seen within the necrosis. By immunohistochemical stains, the lymphoid cells are mostly T lymphocytes that are positive for CD3 (Fig. 3) with no apparent marker expression. CD30 is positive in scattered immunoblasts. The residual of B lymphocytes are positive for CD20 (Fig. 4) and other B-cell markers with no apparent marker expression. Ki-67 is positive in approximately 70% of cells. The histiocytes are highlighted by CD163 and myeloperoxidase. Scattered plasma cells are present and highlighted by CD138. Viral immunostains for EBV, HSV-1, HSV-2 and CMV are all negative. Flow cytometry is negative for monotypic B or T cells. T-cell receptor gene rearrangement showed no clonal T-cell receptor rearrangement. These features were consistent with the diagnosis of Histocytic necrotizing lymphadenitis. The patient was discharged home on in good condition.

## Discussion

Kikuchi's disease (KD) also known as Kikuchi-Fujimoto disease (KFD), or histiocytic necrotizing lymphadenitis was first described in 1972 independently by Kikuchi and Fujimoto et al. [1], [2]. Initially all reports of Kikuchi-Fujimoto disease came from Japan but subsequently it has been reported from Europe, America, Asia and Middle East [3], [4], [5], [6], [7]. Kikuchi-Fujimoto disease is a benign self-limited condition of unknown etiology which usually presents with cervical lymphadenopathy or fever of unknown origin[8]. The disease is rare and so is frequently not included in the differential diagnosis at presentation. There are no specific laboratory tests available for the diagnosis of Kikuchi-Fujimoto disease and diagnosis can be made only after histologic examination of lymph node biopsy. KFD remains an enigmatic disease, not only for its rarity and non-specific manifestations, but also because its etiology and pathogenesis remain unclear and there are no clear diagnostic criteria. Even though it is a benign condition, correct diagnosis is fundamental to rule out other causes of lymphadenopathy. Despite the self-limited nature of this disease, biopsy should be performed to exclude more serious conditions such as lymphoma, tuberculous adenitis, and systemic lupus erythematosus [4]. Organisms such as Epstein-Barr virus, cytomegalovirus, varicella-zoster virus, human herpes virus-6, human immunodeficiency virus, Yersinia enterocolitica, and Toxoplasma gondii have been linked to this condition, but no convincing causal relationship has been identified [9]. An association between KFD and autoimmune disorders has been shown, including SLE, mixed connective tissue disease, antiphospholipid antibody syndrome, and scleroderma [10], [11]. Imamura et al. reported that electron microscopy has often revealed tubuloreticular structures in histiocytoid cells of lymph node lesions of patients with KFD, and they hypothesized that KFD might reflect a self-limited SLE-like autoimmune condition induced by virus-induced transformed lymphocytes [12]. One study suggested that KFD might represent an exuberant T cell-mediated immune response in people genetically susceptible to a variety of nonspecific stimuli because some HLA class II genes were more frequent in patients with KFD [13].

Lymphadenitis occurring after administration of a vaccine is called post-vaccinal lymphadenitis [14]. It is a reactive response to the vaccination and can be caused by a variety of vaccines against smallpox [15], influenza [16], varicella zoster, BCG, and pneumococcal vaccine [15]. KFD following vaccination has been very rarely reported following human papilloma virus and influenza vaccines [16], [17]. Few case reports have linked KFD in association with SARS CoV2 infection[18], [19]. Several cases of lymphadenopathy following COVID 19 vaccination. The median days of LAP presentation after the first and second dosages of COVID-19 vaccination, were 12 and 5 days, respectively. The sites most involved were the axillary and cervical lymph nodes. It has reported with Pfizer-BioNTech (n = 30, 44.1%), Moderna (n = 17, 25%), and Oxford-AstraZeneca vaccines [20], [21], [22]. To our knowledge there have been no reported case of Kikuchi-Fujimoto disease following SARS CoV2 vaccination. How the vaccine may lead the development of KFD disease is unknown, however it might induce it because viral or other antigens in the vaccine could lead to aberrant immune response in vaccine recipients resulting into the development of KFD.

In conclusion, Kikuchi-Fujimoto disease is a benign self-limited condition of unknown etiology. The disease has been reported in association with autoimmune disorders and following vaccination. There are no specific laboratory tests and diagnosis require lymph node biopsy. Including it in the differential diagnosis in the appropriate setting help in excluding other more serious diseases and avoiding subjecting patients to unnecessary treatment. We report this case to alert physicians to this unusual and not previously reported sequalae of SARS CV2 vaccine.

## Consent

We, all the authors acknowledge that our study has been carried out in accordance with The Code of Ethics of the World Medical Association (Declarationof Helsinki) for experiments involving humans. The manuscript is in line withthe Recommendationsfor the Conduct, Reporting, Editing and Publication of Scholarly Work inMedical Journals. We acknowledge that an informed consent wasobtained from the patient. The privacy rights of human subjects were observed.
